# Supplementary material for: Analysis of attacking styles and goal-scoring in the 2021/22 Women’s Super League
Source: PLoS One. 2025 Feb 6;20(2):e0318929. doi: 10.1371/journal.pone.0318929 (PMC11801634; doi:10.1371/journal.pone.0318929)
Supplement: S1 Table — (DOCX) [file pone.0318929.s001.docx]

**Table A.** Categorisation of attack styles from the selected KPIs and relevant action variables, with relevant evidence from literature used to develop the action variable conditions pertaining to each attack style.

| **Key Performance Indicators** | | | | | | | | | | | | | | | | **Type of Attack** |
| --- | --- | --- | --- | --- | --- | --- | --- | --- | --- | --- | --- | --- | --- | --- | --- | --- |
| **Type of start** | | **Defensive organisation** | | **Type of passes** | | **No. of passes** | | **% of penetrative passes** | | **Starting zone of attack** | | **Width of attack** | | **Duration of attack** | |  |
| ***Action variables*** | ***References*** | ***Action variables*** | ***References*** | ***Action variables*** | ***References*** | ***Action variables*** | ***References*** | ***Action variables*** | ***References*** | ***Action variables*** | ***References*** | ***Action variables*** | ***References*** | ***Action variables*** | ***References*** |  |
| Regain / Restart | [1–3] | Balanced | [1, 2] | Short / Mixed | [1, 2, 4] | 4-6 / 7+ | [2, 4–6] | Low % of Penetrative / Medium % of Penetrative | [1, 2] | Defensive / Central / Attacking | [4] | Low / High | [2, 4] | 9-15 seconds / 16 + seconds | [2, 4] | **Combinative organised** |
| Regain / Restart | [1, 2, 7] | Balanced | [2, 7] | Long | [1, 2, 6, 7] | 0-3 | [1, 2, 7] | High % of Penetrative | [1, 2] | Defensive / Central | [4] | Low | [2, 4] | 0-8 seconds | [2, 6] | **Direct organised** |
| Regain / Restart | [2, 7] | Balanced | [2, 7] | Short / Mixed | [2, 7] | 0-3 | [2, 7] | High % of Penetrative | [2, 7] | Defensive / Central / Attacking | [4] | High | [4] | 0-8 seconds | [2, 7] | **Fast**  **organised** |
| Regain | [1, 2, 7, 8] | Balanced / Unbalanced | [2, 7] | Short / Mixed | [4] | 0-3 /  4-6 | [4, 6] | High % of Penetrative | [1, 2, 7] | Defensive / Central / Attacking | [4] | Low | [2, 7] | 0-8 seconds | [1, 2, 4, 8] | **Counter**  **attack** |
| Set Play | [9–11] | NA |  | Short / Medium/ Long | [9, 10, 12] | NA |  | NA |  | Central / Attacking | [10] | NA |  | NA |  | **Set play** |

**References**

1. González-Ródenas J, López Bondía I, Calabuig Moreno F, Aranda Malavés R. Tactical indicators associated with the creation of scoring opportunities in professional soccer. Cultura_Ciencia_Deporte. 2015 Nov 1;10(30):215–25.

2. Aranda R, González-Ródenas J, López-Bondia I, Aranda-Malavés R, Tudela-Desantes A, Anguera MT. “REOFUT” as an Observation Tool for Tactical Analysis on Offensive Performance in Soccer: Mixed Method Perspective. Front Psychol. 2019 Jun 28;10.

3. González-Ródenas J, López-Bondia I, Aranda-Malavés R, Tudela Desantes A, Sanz-Ramírez E, Aranda Malaves R. Technical, tactical and spatial indicators related to goal scoring in European elite soccer. Journal of Human Sport and Exercise. 2019;15(1).

4. Sarmento H, Clemente FM, Harper LD, Costa IT da, Owen A, Figueiredo AJ. Small sided games in soccer – a systematic review. Int J Perform Anal Sport. 2018 Sep 3;18(5):693–749.

5. Gonzalez-Rodenas J, Lopez-Bondia I, Calabuig F, Pérez-Turpin JA, Aranda R. The effects of playing tactics on creating scoring opportunities in random matches from US Major League Soccer. Int J Perform Anal Sport. 2015 Dec 3;15(3):851–72.

6. Papadopoulos S, Papadimitriou K, Konstantinidou X, Ourania M, Pafis G, Papadopoulos D. Factors Leading to Goal Scoring in the Spanish and Italian Soccer Leagues. Sport Mont. 2021 Feb 1;19(1):13–8.

7. González-Rodenas J, Aranda-Malavés R, Tudela-Desantes A, de Matías-Cid P, Aranda R. Different Pitch Configurations Constrain the Playing Tactics and the Creation of Goal Scoring Opportunities during Small Sided Games in Youth Soccer Players. Int J Environ Res Public Health. 2021 Oct 6;18(19):10500.

8. Tenga A, Holme I, Ronglan LT, Bhar R. Effects of match location on playing tactics for goal scoring in Norwegian professional soccer. J Sport Behav. 2010;33(1).

9. López Bondía I, González-Rodenas J, Calabuig Moreno F, Pérez-Turpin JA, Aranda Malavés R. Creación de ocasiones de gol en fútbol de élite. Diferencias tácticas entre Real Madrid CF y FC Barcelona (Creating goal scoring opportunities in elite soccer. Tactical differences between Real Madrid CF and FC Barcelona). Retos. 2017 Mar 16;(32):233–7.

10. Gonzalez-Rodenas J, Lopez-Bondia I, Calabuig F, Pérez-Turpin JA, Aranda R. Creation of goal scoring opportunities by means of different types of offensive actions in US major league soccer. Human Movement. 2017;2017(5):106–16.

11. González-Rodenas J, Martínez López A, Pérez Gay R. Effects of contextual and tactical dimensions on the creation of goal-scoring opportunities in U12 and U13 elite Spanish soccer teams. Int J Sports Sci Coach. 2024 Feb 28;19(1):202–13.

12. González-Ródenas J, López Bondía I, Calabuig Moreno F, Aranda Malavés R. Tactical indicators associated with the creation of scoring opportunities in professional soccer. Cultura_Ciencia_Deporte. 2015 Nov 1;10(30):215–25.
