# Supplementary material for: Analysis of attacking styles and goal-scoring in the 2021/22 Women’s Super League
Source: PLoS One. 2025 Feb 6;20(2):e0318929. doi: 10.1371/journal.pone.0318929 (PMC11801634; doi:10.1371/journal.pone.0318929)
Supplement: Attachment — Submitted filename: Response to Reviewers.docx [file pone.0318929.s002.docx]

**Analysis of attacking styles and goal-scoring in the 2021/22 Women’s Super League.**

**Revision 1**

*We (authors) are extremely grateful for the time and efforts of the reviewers in providing feedback to improve the manuscript.*

| **Reviewer Number** | **Comment Ref No.** | **Initial Line Number/s** | **Reviewer Comments** | **Author Comments** |
| --- | --- | --- | --- | --- |
| Associate Editor | E_1_ | NA | Please ensure that your manuscript meets PLOS ONE's style requirements, including those for file naming. | The format of the manuscript has been improved to align with the PLOS One styling requirements (key changes performed on font sizes). |
|  | E_2_ |  | Please include your full ethics statement in the ‘Methods’ section of your manuscript file. | Relevant ethics statement has been strengthened in the manuscript (see under section 2.2). |
|  | E_3_ |  | We note that you have indicated that there are restrictions to data sharing for this study. For studies involving human research participant data or other sensitive data, we encourage authors to share de-identified or anonymized data. | We apologise for the error from our end when responding to this prompt in the article submission portal. Subsequently, we have made all data pertaining to the paper available on the Open Science Framework. The relevant file can be accessed using the below link.  <https://doi.org/10.17605/OSF.IO/RQUVD> |
|  | E_4_ |  | Please review your reference list to ensure that it is complete and correct. | The reference list has been reviewed and we confirm that it is complete and accurate. |
| Reviewer 1 | R_1_ | Line 41-43 | Insert references to show that your argument is based on empirical evidence. | Added references to strengthen the argument (Cootes, 2023; Okcic, 2023; Culvin, 2019). |
|  | R_2a_ | Line 105 | If you say previous research, insert references. | The article by de Jong et al. (2020) outlines the limited research within women’s football and discusses the need for practitioners to use male focused research. Therefore, this reference has been used to support this judgement. |
|  | R_2b_ | Line 113 | Although the authors intent to close the gap in the literature, but it is important to provide a strong justification of the study. | Few sentences have been added towards the end of the Introduction to strengthen the justification for the study. |
|  | R_2c_ | Line 149 | Consider rephrasing the statement below: “Finally, once the identification KPIs and operational definitions was completed and their validity was established within the literature, they were presented to a panel of experts who further asserted the validity and relevance of the KPI, action variables and categorisation of the attack styles from them.” | Sentence has been reworked within the manuscript. |
|  | R_2d_ | Throughout manuscript | Further, some words are capitalised unnecessary (e.g., Set Play). | All capitalised words were re-examined and those deemed unnecessary were amended. Thereby, consistent use of sentence case has been established. For instance, ‘Set Play’ was changed to ‘set play’ and the capitalisation of terms used for the other attack styles were also changed (e.g. ‘Combinative Organised attack’ changes to ‘combinative organised attack’). |
|  | R_3_ | Line 201 | I think your study is observational. | We completely acknowledge this comment by the reviewer and the reasons behind it. However, as specified in the Introduction, since there exists a current gap in granular tactical analysis of women’s football, our core aim was to undertake an initial descriptive examination of attacking styles which resulted in a shot on target that could potentially guide future research. While we acknowledge that descriptive studies don’t typically test a hypothesis, the inferential analysis undertaken in the study was done to explore if there is a potential relationship between the key variables for the selected WSL season, that may warrant deeper examination in the future (e.g., considering more representative sample of WSL seasons with a clear observational design). Subsequently, the inferential analysis was done as an initial step to examine the viability of generating a clear hypothesis, which could then lead to stronger observational research designs in the future, capable of generating key analytical judgements (e.g., causality assessments by estimating counterfactuals). We apologise for not making this point explicit. Accordingly, the relevant sentence has been adjusted slightly to clarify this point and we hope that the reviewer is happy for us to retain this aspect in the manuscript. |
|  | R_4_ | Line 245 | Line 206: Consider removing this sentence, “Afterwards, bar charts were used to visualise the descriptive results.” | This sentence has been removed from manuscript. |
|  | R_5_ | Lines 313- 346 | Lines 268-281: There are no references to support or contrast your findings. | We completely acknowledge this comment by the reviewer. However, in our writing style, first paragraph of the Discussion has been used to summarise key findings from the study to help articulate the key sub-sections in the Discussion. Thereby, the succeeding paragraphs critically examines each finding from the study (that have been stated in the first paragraph of the Discussion) in relation to key contemporary literature. Consequently, we hope that the reviewer is happy for us to retain this flow in the writing. |
